# Combining distribution modelling and phylogeography to understand present, past and future of an endangered spider

**DOI:** 10.1186/s12862-024-02295-2

**Published:** 2024-08-05

**Authors:** Filippo Milano, Gabriele Casazza, Andrea Galimberti, Davide Maggioni, Marco Isaia

**Affiliations:** 1 Department of Life Sciences and Systems Biology, University of Turin, Turin, 10123 Italy; 2 Department of Earth, Environmental and Life Sciences, University of Genoa, Genoa, 16132 Italy; 3 Department of Biotechnology and Biosciences, University of Milano-Bicocca, Milan, 20126 Italy; 4 National Biodiversity Future Centre, Palermo, 90133 Italy; 5 Marine Research and High Education (MaRHE) Center, University of Milano-Bicocca, Faafu Magoodhoo, 12030 Republic of Maldives

**Keywords:** Alpine spiders, Climate change, Genetic diversity, IUCN, Long-term stability hypothesis, mtDNA, Pleistocene glaciations, Species distribution model, *Vesubia jugorum*, Wolf spiders

## Abstract

**Background:**

Understanding how endangered species respond to climatic changes is fundamental for their conservation. Due to its restricted geographic range, its sensitivity to the ongoing global warming and its continuing decline, the Southwestern-Alpine endemic wolf spider *Vesubia jugorum* is currently classified as Endangered in the IUCN Red List. Here, we combined species distribution modelling (SDM) and phylogeographic inference to describe the present, the past and the future of this species in light of the mtDNA genetic structure of extant populations.

**Results:**

Phylogenetic and network analyses show a high level of genetic differentiation and a strong genetic structure of the populations, likely explicable by a long history of isolation and survival in separate refugia. The SDM projection into past climatic conditions supports these results by showing a smaller distribution range compared to present, mostly restricted to the Maritime and Ligurian Alps, which possibly served as main refugium. Future forecast shows a significant shift in the bioclimatic range towards higher altitudes and latitudes, with a drastic decrease of habitat suitability in the central and south-eastern parts of the range, with consequent general loss of haplotype diversity.

**Conclusion:**

SDM and phylogeographic inference support the hypothesis that the current distribution and the genetic structure of the extant populations mirror the survival in situ of *Vesubia jugorum* across repeated glacial and interglacial phases, in line with the ‘*long-term stability hypothesis*’. Future predictions show a significant shift in the bioclimatic range that *V. jugorum* will be likely unable to track, with profound impact on its long-term survival and its genetic diversity. Our considerations have implication for conservation genetics, highlighting the pivotal role of the transboundary protected areas of the SW-Alps in promoting conservation efforts for this species.

**Supplementary Information:**

The online version contains supplementary material available at 10.1186/s12862-024-02295-2.

## Introduction

Climatic changes strongly influence the distribution and the evolution of species in space and time. Quaternary glaciations, in particular, induced multiple episodes of expansion, contraction and shift of the species’ ranges in the Alps [1], shaping their current distributional patterns and genetic structure, and generating specific areas of endemism [1–3]. Similarly, future warming scenarios are expected to influence the distribution range of a great number of alpine species [4]. High-mountain habitats are expected to be particularly vulnerable to temperature variations, with warming rates approximately doubling the global average [5]. The rapid increase in temperature, and the associated changes in climatic suitability, will prompt latitudinal and altitudinal shifts, resulting in a reduction of range size for mountaintop and dispersal-limited species [6, 7].

The “Expansion-Contraction” model has been proposed to describe the responses of organisms to Pleistocene climate change [3, 8–10], and proved to be useful for predicting the impact of future climate change on species [11]. Accordingly, the majority of studies on alpine species suggest that small populations survived in southern glacial refugia during cold periods, and re-populated higher latitudes through northward range expansions during postglacial warming (‘*post-glacial expansion hypothesis*’) [8–13]. Such a cyclic climatic shift throughout the Pleistocene implied the repeated fragmentation and isolation of populations in glacial refugia, with strong effects on the genetic structure of the species [3, 8, 9, 12, 13]. Consequently, populations persisting in glacial refugia have relatively long and stable demographic history, resulting in higher levels of genetic diversity when compared to populations established in recently colonized areas [14].

However, the “Expansion-Contraction” model might not fully explain the variety of responses of species to climatic variations [15]. It has been proposed that during glacial periods cold-adapted species may have expanded their range in ice-free areas and contracted their distribution as temperature increased through time [16, 17]. According to this ‘*post-glacial contraction hypothesis*’, the Alps served as high-altitude refugial areas during the warm interglacial periods.

Alternatively, some species may have maintained stable populations where the effects of Late Quaternary glaciations were less dramatic and survived in situ via short altitudinal shifts (‘*long-term stability hypothesis*’) [16, 18, 19]. Accordingly, the persistence of species throughout glacial-interglacial appears to be driven by both local and landscape heterogeneity, namely high microhabitat diversification and high landscape diversity [20].

Due to the mild effects experienced during the Quaternary glaciation, the SW-Alps acted as refugium for a remarkable number of species [16, 18, 19, 21–24]. Indeed, the Southwestern-Alpine refugial area is regarded as one of the major hotspots of biodiversity in Europe, characterized by high levels of endemism and by the presence of divergent intraspecific phylogeographic lineages [18, 25]. The impact of past and future climatic conditions and the role of refugia in range dynamics are well documented for endemic plants of the SW-Alps (e.g., [18, 19, 21–27]), but have been rarely considered in high-mountain animal species.

In this study, we integrated information from different analytical methods to investigate the present-day population structure of *Vesubia jugorum* (Simon, 1881), an alpine endemic wolf spider restricted to the SW-Alps, and to infer the effect of past and future climate change on the species’ distribution and genetic diversity. These results may provide key information on the importance of the network of protected areas in SW-Alps for the long-term persistence and conservation of this endangered endemic species.

More precisely, we investigated how: 1) past climatic changes have played a key role in shaping the current geographic distribution and genetic structure of the populations of *V. jugorum*; 2) ongoing climate change will impact the amount of suitable habitat and genetic diversity of this species. 

## Material and methods

### Model species

**Fig. 1:**
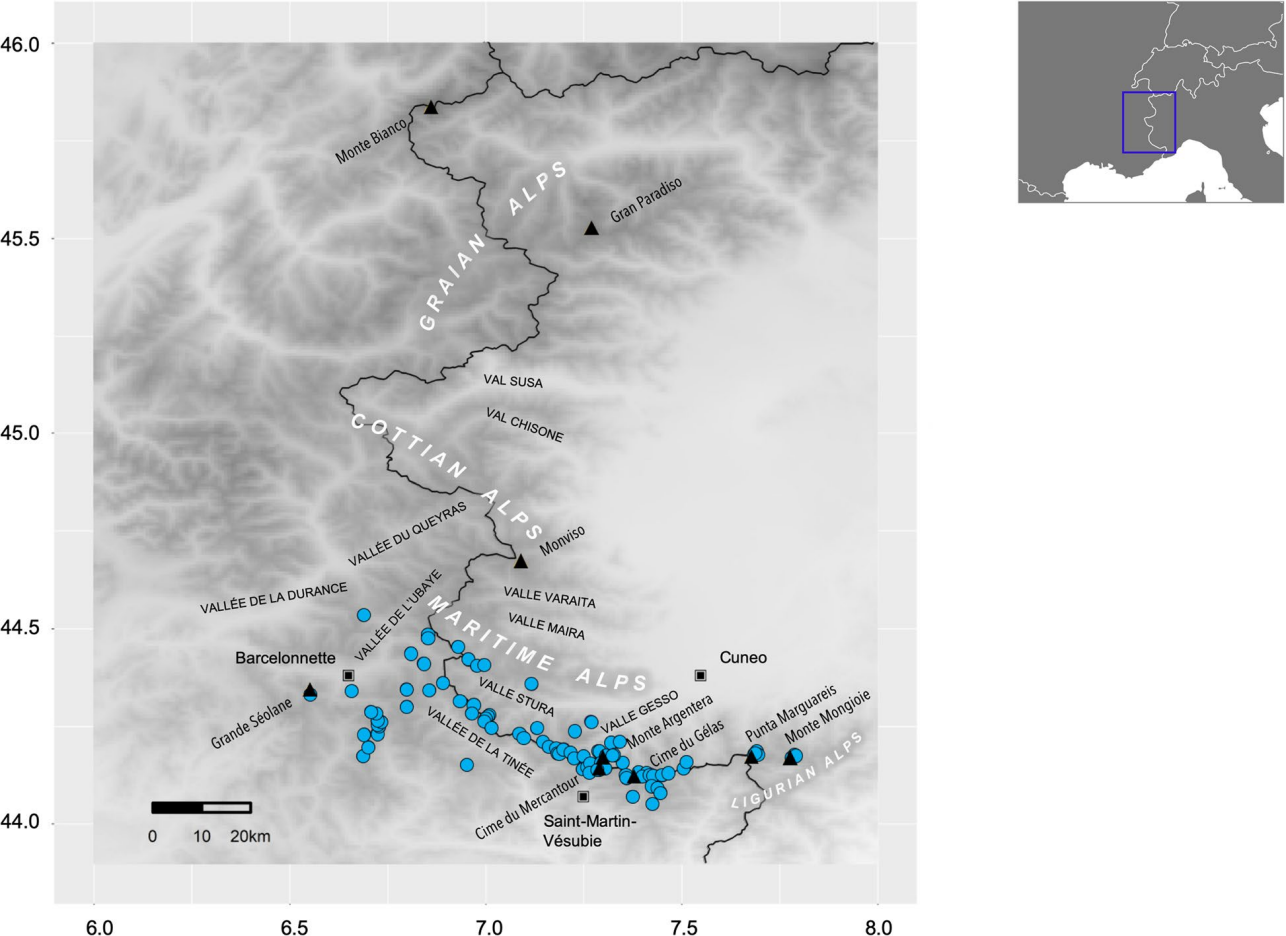
Current occurrences (blue points) of *Vesubia jugorum*. The map was created using the ‘ggplot2’ R package on a digital elevation model for the SW-Alps

*Vesubia jugorum* (Araneae, Lycosidae) is a wolf spider inhabiting high-altitude habitats, such as rocky debris, boulder fields and Alpine screes mostly above 2,300 m [28–31]. Its distribution range spans across the SW-Alps (Fig. 1), at the French-Italian border, encompassing the Ligurian and Maritime Alps, the southern part of the Cottian Alps, and the Provence Alps [30, 31]. The small geographic range, the habitat specialisation and the apparent lack of aerial dispersal [30], suggest a low dispersal ability for this species. Previous preliminary research based on species distribution modelling demonstrated the sensitivity of this species to global warming, with an expected significant reduction of its future bioclimatic range [32]. Because of its small distributional range and the projected continuing decline, *V. jugorum* has been classified as Endangered according to the criteria of the International Union for Conservation of Nature (IUCN) [33]. Accordingly, a long-term monitoring programme, based on the relationship between habitat quality and functional traits [29, 30], has been designed for detecting changes in populations and for evaluating the ongoing impact of climate change on the species survival.

### Genetic analyses

#### Sampling and data acquisition

**Fig. 2:**
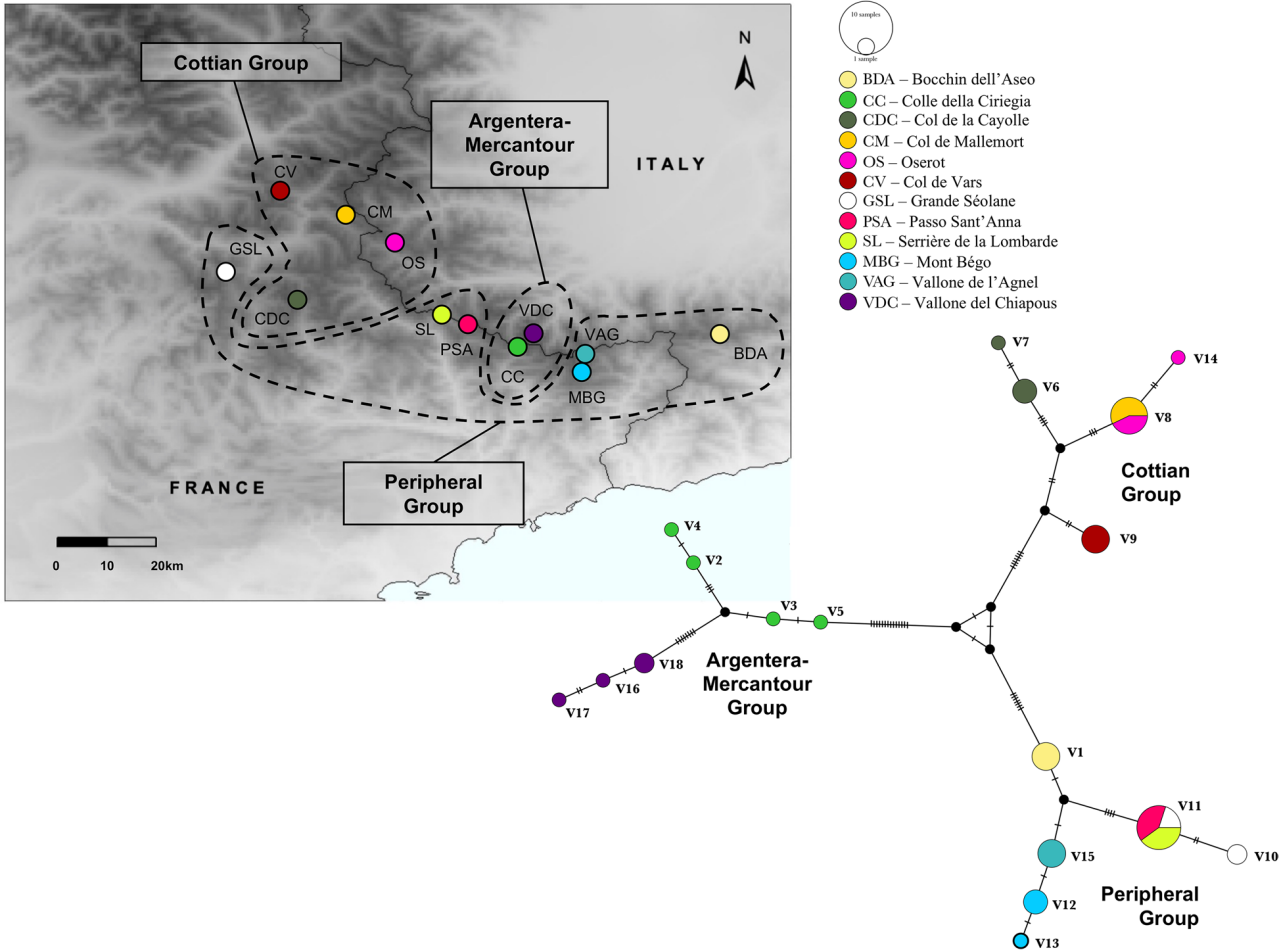
Sampling localities and haplotype network of the investigated populations of *Vesubia jugorum*. Codes in the map indicate localities (see legend), alphanumeric codes in the networks refer to haplotypes. The size of each circle is proportional to the number of sampled individuals with each haplotype (see scale above the legend). Map was created using the ‘ggplot2’ R package on a digital elevation model for the SW-Alps

Specimens of *Vesubia jugorum* were hand-collected in summer 2016, 2017 and 2018 at 12 localities scattered across its distributional range in the SW-Alps (Fig. 2). Specimens were stored at -20 °C after collection. For each locality, four specimens were used for genetic analyses, resulting in genetic data from 48 individuals (Table 1). The toponyms and classification of the different sectors and sub-sectors of the Alps follows the partition of the Alpine chain (SOIUSA; [34]).

**Table 1:** Samples included in the analyses, with information on the sampling locality, coordinates in decimal degrees (Datum: WGS 84), altitude, haplotype and group coding, and sex

Sample	Locality	Coordinates	Altitude (m)	Haplotype	Group	Sex
BDA1	Italy: Bocchin dell'Aseo	44.1745 N 7.7926 E	2295	V1	Peripheral	Female
BDA3	Italy: Bocchin dell'Aseo	44.1745 N 7.7926 E	2295	V1	Peripheral	Female
BDA4	Italy: Bocchin dell'Aseo	44.1745 N 7.7926 E	2295	V1	Peripheral	Female
BDA5	Italy: Bocchin dell'Aseo	44.1745 N 7.7926 E	2295	V1	Peripheral	Female
CC1	Italy: Colle della Ciriegia	44.1418 N 7.2831 E	2543	V2	Argentera-Mercantour	Female
CC2	Italy: Colle della Ciriegia	44.1418 N 7.2831 E	2543	V3	Argentera-Mercantour	Female
CC3	Italy: Colle della Ciriegia	44.1418 N 7.2831 E	2543	V4	Argentera-Mercantour	Female
CC5	Italy: Colle della Ciriegia	44.1418 N 7.2831 E	2543	V5	Argentera-Mercantour	Female
CDC2	France: Col de la Cayolle	44.2625 N 6.7323 E	2459	V6	Cottian	Juvenile
CDC5	France: Col de la Cayolle	44.2625 N 6.7323 E	2459	V6	Cottian	Juvenile
CDC6	France: Col de la Cayolle	44.2625 N 6.7323 E	2459	V6	Cottian	Female
CDC7	France: Col de la Cayolle	44.2625 N 6.7323 E	2459	V7	Cottian	Female
CM1	France: Col de Mallemort	44.4749 N 6.8532 E	2560	V8	Cottian	Female
CM2	France: Col de Mallemort	44.4749 N 6.8532 E	2560	V8	Cottian	Female
CM4	France: Col de Mallemort	44.4749 N 6.8532 E	2560	V8	Cottian	Female
CM6	France: Col de Mallemort	44.4749 N 6.8532 E	2560	V8	Cottian	Juvenile
CV2	France: Col de Vars	44.5345 N 6.6894 E	2355	V9	Cottian	Female
CV3	France: Col de Vars	44.5345 N 6.6894 E	2355	V9	Cottian	Male
CV4	France: Col de Vars	44.5345 N 6.6894 E	2355	V9	Cottian	Juvenile
CV5	France: Col de Vars	44.5345 N 6.6886 E	2376	V9	Cottian	Female
GSL1	France: Grande Séolane	44.3318 N 6.5520 E	2520	V10	Peripheral	Juvenile
GSL3	France: Grande Séolane	44.3335 N 6.5512 E	2547	V11	Peripheral	Juvenile
GSL4	France: Grande Séolane	44.3335 N 6.5512 E	2547	V11	Peripheral	Juvenile
GSL5	France: Grande Séolane	44.3335 N 6.5512 E	2547	V10	Peripheral	Female
MBG1	France: Mont Bégo	44.0806 N 7.4459 E	2617	V12	Peripheral	Female
MBG2	France: Mont Bégo	44.0806 N 7.4459 E	2617	V12	Peripheral	Female
MBG3	France: Mont Bégo	44.0806 N 7.4459 E	2617	V12	Peripheral	Female
MBJ1	France: Mont Bégo Bas	44.0811 N 7.4477 E	2565	V13	Peripheral	Juvenile
OS1	Italy: Oserot	44.4052 N 6.9770 E	2500	V8	Cottian	Female
OS2	Italy: Oserot	44.4052 N 6.9770 E	2508	V8	Cottian	Juvenile
OS5	Italy: Oserot	44.4052 N 6.9770 E	2508	V14	Cottian	Juvenile
OS6	Italy: Oserot	44.4052 N 6.9770 E	2508	V8	Cottian	Juvenile
PSA1	Italy: Passo Sant'Anna	44.2220 N 7.0957 E	2396	V11	Peripheral	Female
PSA2	Italy: Passo Sant'Anna	44.2220 N 7.0957 E	2396	V11	Peripheral	Male
PSA3	Italy: Passo Sant'Anna	44.2224 N 7.0951 E	2394	V11	Peripheral	Female
PSA5	Italy: Passo Sant'Anna	44.2220 N 7.09572 E	2396	V11	Peripheral	Female
SL1	France: Serrière de la Lombarde	44.1982 N 7.1604 E	2337	V11	Peripheral	Female
SL2	France: Serrière de la Lombarde	44.1982 N 7.1604 E	2337	V11	Peripheral	Female
SL3	France: Serrière de la Lombarde	44.1982 N 7.1604 E	2337	V11	Peripheral	Female
SL4	France: Serrière de la Lombarde	44.1982 N 7.1604 E	2337	V11	Peripheral	Female
VAG1	France: Vallon de l'Agnel	44.1240 N 7.4527 E	2350	V15	Peripheral	Female
VAG2	France: Vallon de l'Agnel	44.1240 N 7.4527 E	2350	V15	Peripheral	Female
VAG4	France: Vallon de l'Agnel	44.1240 N 7.4527 E	2350	V15	Peripheral	Juvenile
VAG5	France: Vallon de l'Agnel	44.1240 N 7.4527 E	2350	V15	Peripheral	Female
VDC1	Italy: Vallone del Chiapous	44.1769 N 7.3263 E	2307	V16	Argentera-Mercantour	Female
VDC2	Italy: Vallone del Chiapous	44.1769 N 7.3266 E	2318	V17	Argentera-Mercantour	Female
VDC3	Italy: Vallone del Chiapous	44.1783 N 7.3227 E	2324	V18	Argentera-Mercantour	Male
VDC6	Italy: Vallone del Chiapous	44.1769 N 7.3263 E	2307	V18	Argentera-Mercantour	Female

#### DNA extraction, amplification and sequencing

One leg was removed from each specimen for DNA extraction. Whole genomic DNA was extracted using the NucleoSpin® Tissue kit (Macherey-Nagel GmBH) following the manufacturer’s protocol.

Two partially overlapping regions of 625 bp and 1,025 bp of the mitochondrial cytochrome c oxidase subunit I (COI) gene were amplified by polymerase chain reaction (PCR) using the primer pairs LCO1490 (5′-GGTCAACAAATCATAAAGATATTGG-3′; [35])—HCO2183R2 (5′-CCAAAAAATCAAAATARATGYTG-3′; [36]) and C1-J-1751 (5′-GAGCTCCTGACATAGCATTCCC-3′; [37])—C1-N-2776 (5′-GGATAATCAGAATATCGTCGAGG-3′; [38]) respectively.

PCR amplifications were carried out in 12.5 μL reaction volume in a final reaction mix composed of 1 μL of DNA sample with 1.25 μL of dNTPs, 1.25 μL *Taq* buffer, 0.5 μL MgCl_2_, 0.125 μL *Taq* polymerase and 0.125 μL of each primer. PCR amplification included an initial denaturation of 10 min at 94 °C followed by 39 cycles of 30 s denaturation at 94 °C, annealing at 47 °C for 30 s, and extension at 72 °C for 90 s; finally, a final elongation step at 72 °C for 5 min was conducted. The final products were purified using ExoSAP-IT™ PCR Product cleanup reagent (Thermo Fisher Scientific) prior to sequencing. PCR products were sequenced bidirectionally at Macrogen, Inc. We used Geneious Prime® 2023.2.1 to assemble the forward and reverse chromatograms and to check for potential errors that result in stop codons of the translated sequences. COI sequences were deposited in GenBank NCBI (see Additional File 2). Sequences were aligned using MAFFT 7.110 [39] with the E-INS-i option, after adding the two outgroups *Pardosa laura* (GenBank accession number: NC025223) and *Lycosa oculata* (GenBank accession number: KC550670).

#### Phylogenetic and genetic structure analyses

Phylogenetic inference was performed using maximum likelihood (ML) and Bayesian inference (BI). ML analyses were conducted using RAxML 8.2.12 [40] specifying a GTR substitution model. Node support levels were obtained from 1,000 non-parametric bootstrap replicates. For BI analyses, the best substitution models and partitions were determined with PartitionFinder 1.1.1 [41], using the Bayesian Information Criterion (partition by codon: pos1 = TrN + I, pos2 = HKY, pos3 = HKY). Before reconstructing the phylogenetic hypothesis, the marginal likelihood estimations of the clock and non-clock models were obtained with MrBayes 3.2.7 [42], resulting in the strict clock model being highly supported (2log(B_12_) = 548.4) [43]. BI was performed with the software BEAST 1.8.2 [44], setting a coalescent tree prior and a strict clock. Since no reliable fossil records or well-dated biogeographic events were available for calibrating the tree, the substitution rate was set to 0.01679 substitutions per million years, as obtained from Piacentini and Ramirez [45] for the COI gene in the family Lycosidae. Three independent replicate analyses were run for 100 million generations each, sampling every 10,000th and were then combined using LogCombiner 1.8.2 [44], setting a burn-in of 25%. Stationarity and unimodal posterior distribution of the parameters were checked with Tracer 1.6 [46] and the maximum clade credibility tree was obtained using TreeAnnotator 1.8.2 [44].

To assess if the mtDNA supported the presence of cryptic species among the investigated populations, we applied a DNA-based species delimitation approach. Specifically, we used the single-threshold Generalized Mixed Yule Coalescent (st-GMYC) method [47] using the packages ‘apes’ [48] and ‘splits’ [49] in R, using the ultrametric Bayesian tree as input.

Measures of population genetic diversity (i.e., n haplotypes, haplotype diversity, nucleotide diversity) of each sampling locality and clade were calculated with DnaSP 6 [50]. To visualize the correspondence between genetic diversity and geographic provenance of samples, a median-joining haplotype network was built using the software PopART 1.7 [51], with haplotypes coloured according to sampling localities. An analysis of molecular variance (AMOVA) was also performed with Arlequin 3.5 [52, 53] with 10,000 permutations, grouping sequences by clade and sampling localities within clade.

Pairwise genetic distances within and between sampling localities were calculated as average % uncorrected *p*-distances and their standard deviation in MEGA X [54] and plotted as a heatmap with the ‘ggplot2’ package [55] in the R Statistical Software [56]. Similarly, genetic distances were calculated within and between the main clades inferred from the phylogenetic reconstruction, haplotype network reconstruction and AMOVA. Pairwise genetic distances between the samples were plotted against their geographic distance, the latter calculated from sampling coordinates using the packages ‘geosphere’ and ‘ggplot2’ [55, 57]. A Mantel test [58] using the package ‘vegan’ [59] was then performed to test for a significant correlation between genetic and geographic distances.

Finally, we used Bayesian skyline plots (BSPs) to infer the demographic history of the species. Since BSPs proved to be sensitive to population structure, we used this method to examine the demographic changes in effective population size through time for the whole species and for the clades obtained with phylogenetic analyses separately, [60]. BSPs were obtained with BEAST 2.7.3 [61], setting a strict clock, and the same substitution rate and models used for the phylogenetic reconstruction. Analyses were run for 100 million generations each, sampling every 10,000th and setting a burn-in of 25%. Tracer 1.6 [46] was used to check stationarity and unimodal posterior distribution of the parameters, and to generate plots for each analysis.

#### Ecological niche modelling

We conducted all analyses in the R Statistical Software [56]. To maximise reproducibility and transparency of the species distribution models, we followed the ODMAP (Overview, Data, Model, Assessment and Prediction) protocol [62] (Additional File 1).

We recovered 107 presences of *Vesubia jugorum* (Fig. 1) based on the available data in literature [29, 30]. To avoid overrepresentation of certain regions due to sampling biases, we performed spatial thinning using the function *thin* in the ‘red’ R package [63]. We thinned occurrences through 100 iterations, removing records closer than 0.5% of the maximum distance between any two, and keeping as many as possible of the original records. The thinning value was chosen to consider the neighbor occurrences as spatially independent, based on the extent of the distribution range of the model species and on the resolution of the bioclimatic predictors used. We calibrated and projected models within the spatial extent hypothesized to be accessible to the species via long-term dispersal or colonization over its evolutionary history (the ‘M area’, [64]). Considering the distribution range and the low dispersal potential of this species [29, 30], we masked the study area to 43.9 to 46.0° latitude and 6.0 to 8.0° longitude.

We modeled the current distribution range of the species using bioclimatic variables for “near-present” conditions (1970–2000) and elevation data from WorldClim 2 [65], all at a spatial resolution of 30 arc-seconds. To minimise the multicollinearity among variables, we performed a Principal Component Analysis generating new axes that summarized variation in fewer, independent dimensions.

Given the lack of reliable absence data, we constructed presence-background (MaxEnt) models, using the ‘dismo’ R package with default settings [66, 67]. To evaluate model performance, we adopted the Boyce index [68] as implemented in the *ecospat.boyce* function in the ‘ecospat*’* R package [69], an appropriate metric when lacking absence data [70]. We ran 50 bootstrap replicates, retaining a random partition of 20% of the occurrence points from each run, which was used to evaluate the predictive performance. After model validation, in order to provide a comprehensive and accurate understanding of the species-environment relationship, we generated a final model with the whole dataset resulting from thinning as in Mammola et al. [29], and we projected it into near-present conditions to represent the current distribution of the species. We then projected the model into past and future climatic scenarios to estimate variations in the distribution ranges.

We obtained Paleoclimatic data for the Last Glacial Maximum (~ 21,000 years ago) at 2.5 arc-minutes spatial resolution from the Earth System Model based on the Model for Interdisciplinary Research on Climate (MIROC-ESM).

To predict the potential consequences of future climate, we adopted a new set of integrated emission scenarios, combining the Representative Concentration Pathways (RCPs) with specific socioeconomic and technological development, i.e. the Shared Socioeconomic Pathways (SSPs), as discussed in O’Neill et al. [71] and in van Vuuren et al. [72]. The Shared Socioeconomic Pathways are reference pathways describing plausible alternative trends in the evolution of society and ecosystems over a century timescale [73]. We selected a sustainable (RCP2.6, SSP1) and a fossil-fuelled (RCP8.5, SSP5) development scenario. We projected these scenarios in a range of 20-year-period outcome (2021–2040), with a spatial resolution of 30 arc-seconds. Among available Coupled Model Intercomparison Project Phase 6 (CMIP6) climate models, we used the IPSL‐CM6A‐LR climate model [74].

## Results

### Phylogenetic relationships and genetic structure

The final alignment included 1184 bp of the COI mtDNA gene obtained for all the 48 specimens of *Vesubia jugorum* analysed in this work and did not show any internal stop codon or gap.

**Fig. 3:**
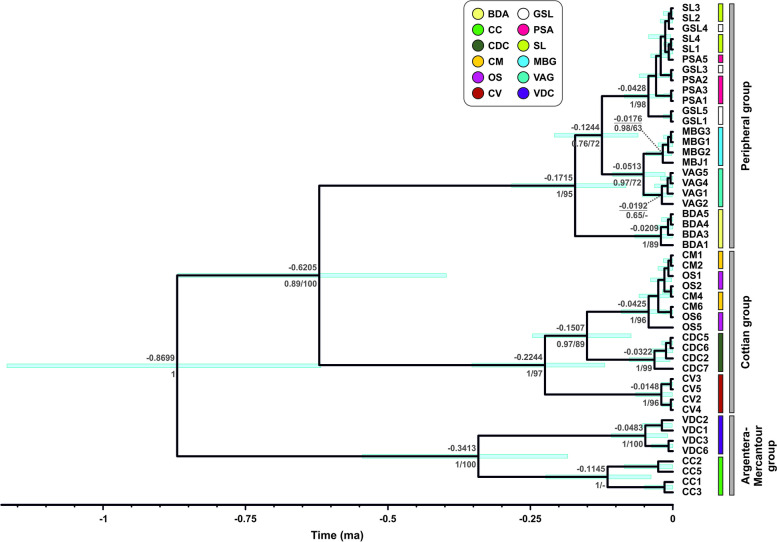
Time-calibrated Bayesian tree based on the COI region of *Vesubia jugorum*. Numbers above nodes show the estimated divergence times, whereas numbers below nodes show Bayesian posterior probabilities and maximum likelihood bootstrap values, respectively. Light blue bars at nodes indicate the 95% HPD confidence intervals of the divergence times. Colored bars at tips represent the sampling localities, as shown in the legend. Abbreviations of the sampling localities are explained in Fig. 2 and in Table 1

Maximum likelihood and Bayesian inference yielded highly similar topologies (Fig. 3). Sequences clustered into three main and well-supported clades. The first clade included two populations from geographical adjacent localities, occurring in the central portion of the Maritime Alps (corresponding to the Argentera-Mercantour Group). A second clade, further divided into three well-supported subclades, included the northern populations (mirroring the Cottian Group). A third clade included the remaining populations from the eastern and central portion of the species’ distribution range, along with the westernmost population of Grande Séolane (corresponding to the Peripheral Group). The estimated time of the diversification of the clades was 0.87 million years ago (95% HPD = 0.62–1.17 million years ago), with the split between the second and the third clades estimated at 0.62 million years ago (95% HPD = 0.4–0.87 million years ago). It must be noted that the estimated times of diversification must be taken carefully due to the large and overlapping 95% HPDs.

The st-GMYC analysis did not reject the null model that all clades originated from a single species (likelihood ratio = 5.0319, *p*-value = 0.08), therefore not supporting the presence of cryptic species.

Across the 12 sampled populations, 18 haplotypes were identified, the majority of them (16 out of 18) being exclusive to a certain geographic locality (Fig. 2). Only haplotypes V8 and V11 were shared between two (i.e., CM and OS) and three (i.e., PSA, SL and the more distant GSL) localities, respectively (Fig. 2). Overall, the haplotype network showed three main groups of related haplotypes, in agreement with the three clades found in the phylogenetic reconstructions, separated by a consistent number of substitutions (i.e., range 24-31; Fig. 2) and geographically corresponding to (i) the Argentera-Mercantour Group (7 haplotypes), (ii) the Cottian Group (5 haplotypes) and (iii) the Peripheral Group (6 haplotypes). The Argentera-Mercantour Group showed the highest haplotype and nucleotide diversity values (i.e., h = 0.964 and π = 0.00666 ± 0.0009), with two subgroups of haplotypes corresponding to two close localities (CC and VDC). Conversely, the other two groups, characterized by haplotypes from nearby localities, showed lower values of haplotype and nucleotide diversity with the lowest values found in the Peripheral Group (Table 2). The AMOVA revealed that most of the genetic variance (76.24%) was ascribable to variation among groups, whereas 20.85% occurred among sampling localities within groups and 2.91% within sampling localities (Additional File 3).

**Table 2:** Genetic indices for each sampling locality, group, and for the whole dataset, with information on number of haplotypes (N), haplotype diversity (h), and nucleotide diversity (π)

Sampling locality	Number of sequences	N	Group	h	π (sd)
Bocchin dell'Aseo (BDA)	4	1	Peripheral	0	0 (0)
Colle della Ciriegia (CC)	4	4	Argentera-Mercantour	1	0.00293 (0.00074)
Col de la Cayolle (CDC)	4	2	Cottian	0.500	0.00088 (0.00047)
Col de Mallemort (CM)	4	1	Cottian	0	0 (0)
Oserot (OS)	4	2	Cottian	0.500	0.00088 (0.00047)
Col de Vars (CV)	4	1	Cottian	0	0 (0)
Grande Séolane (GSL)	4	2	Peripheral	0.667	0.00117 (0.00036)
Passo Sant'Anna (PSA)	4	1	Peripheral	0	0 (0)
Serrière de la Lombarde (SL)	4	1	Peripheral	0	0 (0)
Monte Bego (MBG)	3	1	Peripheral	0	0 (0)
Monte Bego Basso (MBJ)	1	1	Peripheral	0	0 (0)
Vallon de l'Agnel (VAG)	4	1	Peripheral	0	0 (0)
Vallone del Chiapous (VDC)	4	3	Argentera-Mercantour	0.833	0.00147 (0.00053)
Argentera-Mercantour Group	8	7		0.964	0.00666 (0.0009)
Cottian Group	16	5		0.750	0.00467 (0.00057)
Peripheral Group	24	6		0.779	0.00311 (0.00025)
Overall dataset	48	18		0.918	0.01501 (0.001)

**Fig. 4:**
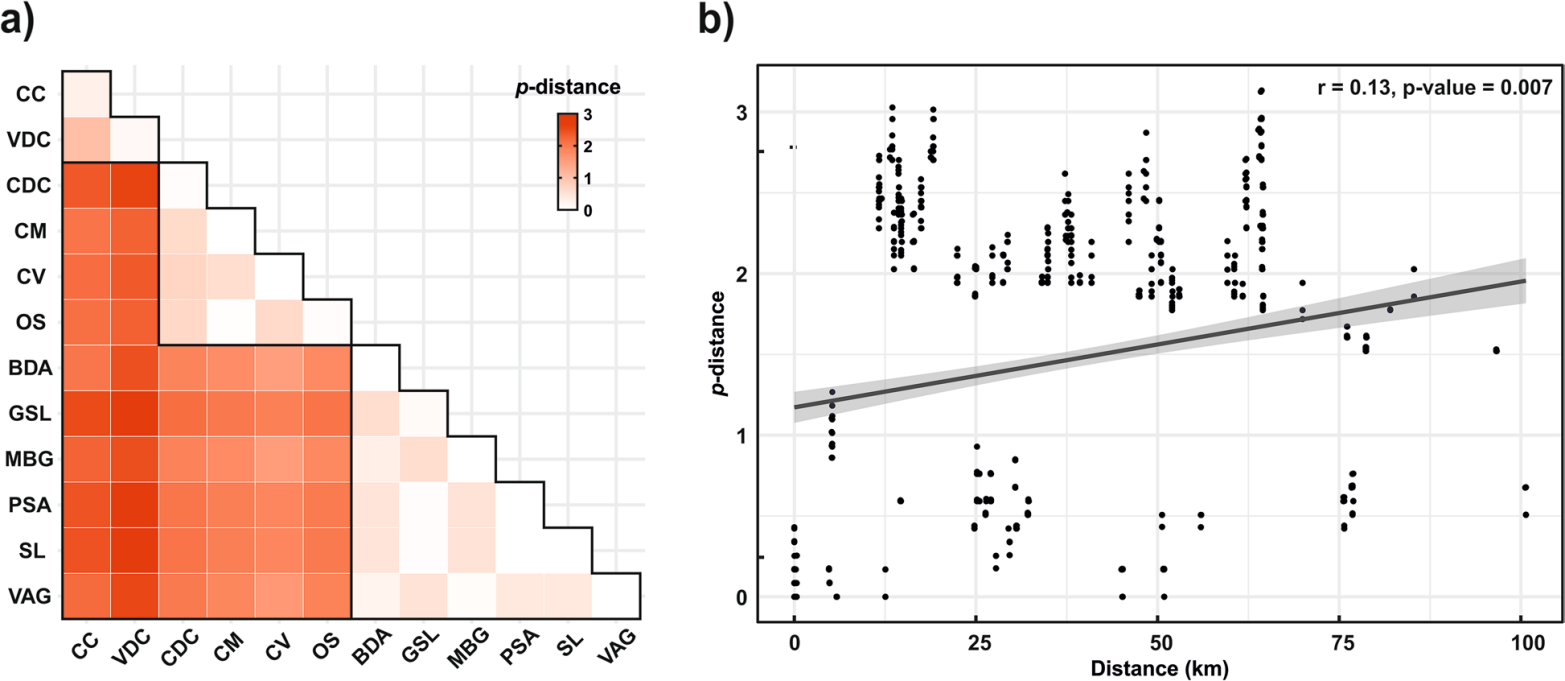
Genetic distances and isolation-by-distance plot in *Vesubia jugorum*. **a** Heatmap showing the pairwise % uncorrected p-distances within and between sampling localities, with darker colours indicating higher distances. Black outlines show the intra-group distances. Abbreviations of the localities are explained in Fig. 2 and in Table 1. **b** Genetic distances plotted against geographic distances, with the interpolation line in red

The overall mean intraspecific distance in *Vesubia jugorum*, calculated as % uncorrected *p*-distance, was 1.5 ± 0.2% and ranged between 0 and 2.9%, with the Argentera-Mercantour Group being most distant from the Peripheral (2.6 ± 0.4%) and the Cottian one (2.4 ± 0.4%) and the average genetic distance between the Peripheral and the Cottian Group being lower (1.9 ± 0.3%) (Fig. 4a and Additional File 4). We found a weak, though significant, positive correlation between genetic and geographic distance among the populations (Mantel test: *r* = 0.13, *p*-value = 0.007) (Fig. 4b).

**Fig. 5:**
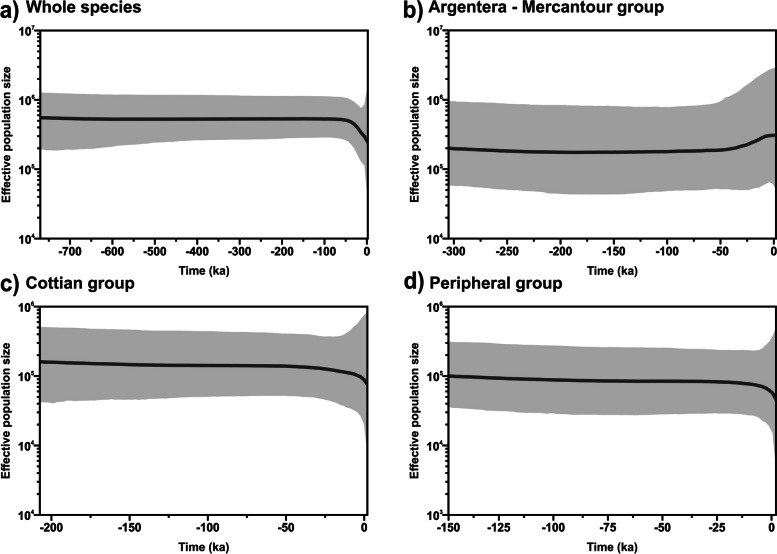
Bayesian skyline plots for *Vesubia jugorum* based on the **a**) overall dataset, **b**) Argentera-Mercantour Group, **c**) Cottian Group, and **d**) Peripheral Group showing effective population size through time. The solid lines indicate the median estimates, whereas the grey areas represent the 95% HPD intervals

Bayesian skyline plot of the whole species pool revealed a constant effective population size, followed by a reduction starting from about 50 kya (Fig. 5a). When analysed separately, the three groups showed different trends, with the Argentera-Mercantour Group slightly increasing from about 25 kya (Fig. 5b) and the Cottian and Peripheral decreasing in the last 25 kya (Fig. 5c and d). With due precautions, the effective population size estimated for these last 25 kya showed an increase in the 95% HPDs.

### Species distribution models and model performance

To generate species distribution models, we kept 84 occurrences after spatial thinning. The first four principal components cumulatively explained around 95% of the overall variance in the dataset (Additional File 5). The Boyce index indicated a high explanatory ability of the distribution models (Boyce index > 0.77; median of the 50 bootstraps).

### Current potential distribution

**Fig. 6:**
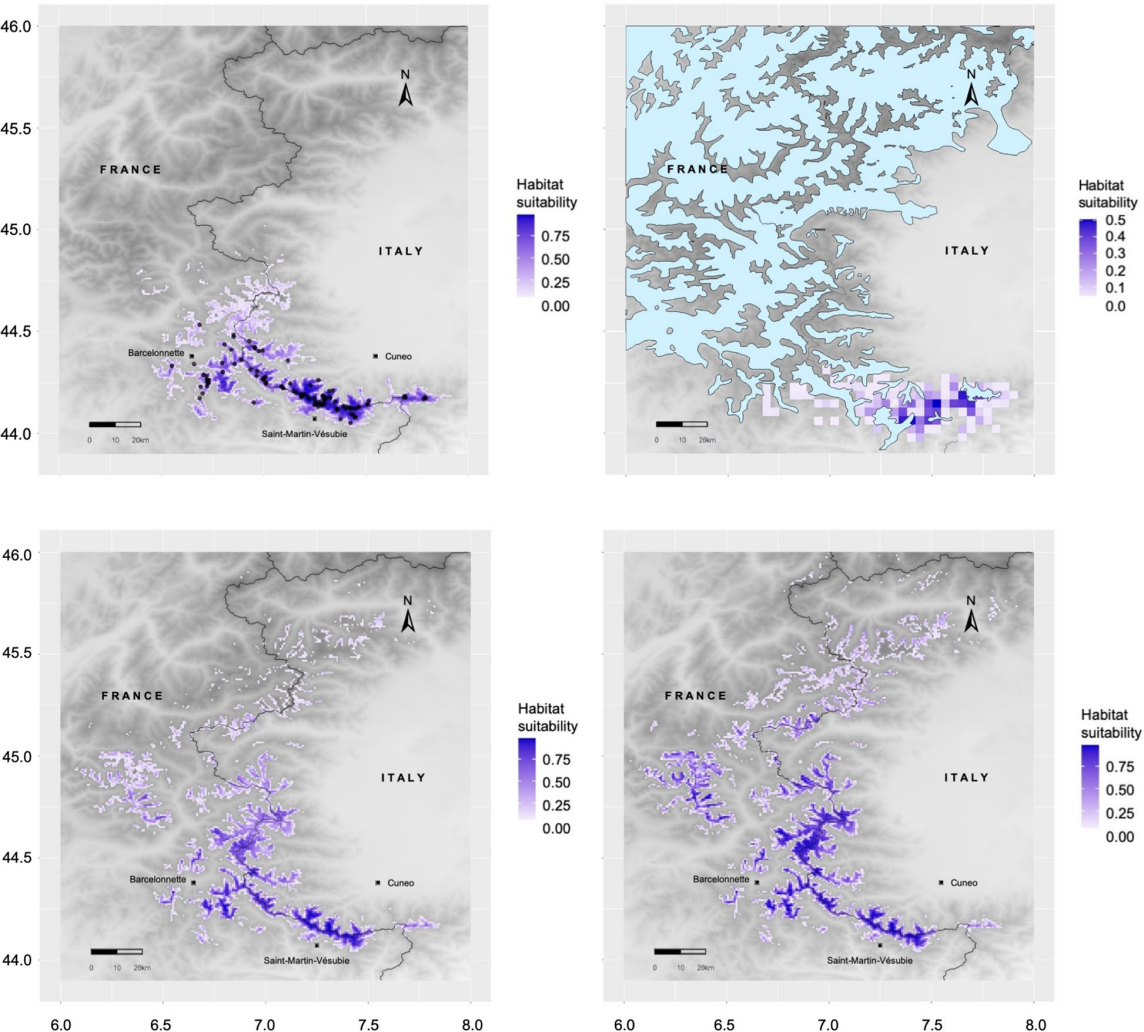
Maps of the bioclimatic suitability for *Vesubia jugorum* at the near-present climate (**a**), during the Last Glacial Maximum (**b**), and in 2021–2040 according to a sustainable (**c**) and a fossil-fuelled (**d**) development scenario. The predicted species distribution is shown in blue in all maps. Black dots in **a**) represent the known localities of the species. Limits of the ice cover in the Last Glacial Maximum [78] are reported for Pleistocene projections as light-blue shapes in **b**). Maps were created using the ‘ggplot2’ R package on a digital elevation model for the SW-Alps

The present-day suitable area estimated by the model is mostly coincident with the known geographic distribution of the species (Fig. 6a). Predictions are congruent with previous modelling exercises on the species [29, 32]. The most suitable and unfragmented area corresponds to the transboundary protected area of Parco Naturale Alpi Marittime in Italy and Parc National du Mercantour in France, encompassing the Argentera-Mercantour Massif at the core of the district of the Maritime Alps. Another suitable, isolated area was predicted in the southernmost part of the species range, in the area corresponding to the Marguareis-Mongioie Massif, in the Ligurian Alps, which is also under the protection of Parco Naturale Alpi Marittime. Additional suitable areas were detected in the southwestern limits of the known distribution, across the Provence Alps, mainly within the borders of Parc National du Mercantour.

Northwards, the predicted range extended beyond the known limit of distribution, in the high Varaita Valley (Italy) and in the northern edge of the high Ubaye Valley (France), across the border between the French departments of Alpes-de-Haute-Provence and Haute-Alps (Fig. 6a). In these regions, the level of habitat suitability was lower compared to the south.

### Past projected distribution

The projection of the potential distribution of *Vesubia jugorum* into the past climatic conditions showed a smaller range compared to present (Fig. 6b). The potential past distribution was found to coincide with areas devoid from glaciers during the Pleistocene. According to the model predictions, the northern portion of the current range was unsuitable during the Last Glacial Maximum, and the suitable areas were restricted to the Argentera-Mercantour and the Marguareis-Mongioie massifs, i.e. the two areas with the highest current suitability, both within the borders of the protected areas of Parco Naturale Alpi Marittime (Italy) and Parc National du Mercantour (France). Several isolated areas of low habitat suitability scattered in the Pleistocenic ice masses appeared along the French-Italian border (Tinée/Stura watershed), and between the departments of Alpes-Maritimes and Alpes-de-Haute-Provence in France (between Var and Verdon Valleys).

### Future projected distribution

Future forecasts, obtained by projecting the habitat suitability under sustainability (SSP1-RCP2.6) and fossil-fuelled development (SSP5-RCP8.5) scenarios, showed significant shifts in the bioclimatic range towards higher altitude and latitudes (Fig. 6c and d).

In both future scenarios, we detected a general decrease in the habitat suitability in the central and south-eastern parts of the range. By contrast, we predicted the appearance of new suitable areas in the northern part of the range.

According to the SSP1-RCP2.6 scenario, a decrease in the suitability was observed all over the current range (Fig. 6c). The reduction in the availability of future suitable areas was higher in the Ligurian Alps, where the remaining patch of suitable habitat is expected to achieve complete isolation. Despite experiencing fewer changes in habitat suitability, we observed a general thinning of the suitable areas in the Maritime and southern Cottian Alps, suggesting possible shifts towards higher altitudes. Conversely, we predicted an increase in habitat suitability in the northern Cottian Alps, with a slight north-westward shift.

On the other hand, the SSP5-RCP8.5 scenario (Fig. 6d) indicated a stronger contraction of the current suitability, with a remarkable north shift of the suitable range reaching the northern Cottian and the Graian Alps. Several areas of currently available suitable habitat in the Maritime Alps were predicted to decrease their suitability, with an increasing isolation of the central current range. The extent of suitable area in the Ligurian Alps was expected to further reduce, as well as the isolated patches in the south-west of the range, in the Provence Alps.

## Discussion

Changes in habitat suitability and species ranges due to climatic variations proved to influence population diversity and current patterns of genetic variation in Alpine endemic species [3, 13, 16, 75–77]. Our results support these findings for *Vesubia jugorum*, suggesting that variations in climate and movements of ice masses during Quaternary glacial cycles [78] concurred in shaping the distributional pattern of this species over time and its intraspecific genetic diversity*.* We interpret the high genetic structuring unravelled by our analyses and the age of internal nodes in the phylogeny as the result of long isolation of populations in refugia [79, 80], enhanced by low dispersal ability and low connectivity of the habitat, further preventing gene flow between populations. Phylogenetic and haplotype network analyses highlighted the presence of three divergent clades, partially corresponding to possible geographical barriers––possibly still persistent––among the sampled populations.

The topography of this region may have represented a primary potential obstacle that directly prevented gene flow among populations. Indeed, the Argentera-Mercantour massif is geologically isolated, characterized by high altitudes (up to 3,300 m) and deep valleys with high differences in height (up to 2,000 m) between the valley floors and the mountain tops. Populations of the Argentera-Mercantour Group, restricted to the central portion of the massif, may have remained isolated from the surrounding populations of the Peripheral Group, promoting accumulation of genetic diversity. At the same time, the strong altitudinal gradients of Stura and Ubaye valleys, coupled with the presence of large valley glaciers during glacial and early-interglacial periods [78], formed effective strong barriers, justifying the observed loss of genetic continuity between the Peripheral and the Cottian groups.

Tentatively, the three main lineages possibly persisted during repeated glacial and interglacial periods in situ within multiple refugia situated in the SW-Alps, experiencing some contractions during the glacial periods (‘in situ survival’ sensu [79]). Genetic differentiation between populations with relatively constant population size, as observed for *V. jugorum*, suggests random genetic drift and low gene flow among populations, as also supported by the inferred missing haplotypes recovered in our haplotype network. The limited dispersal ability of *V. jugorum* and the isolation of mountain peaks due to deep valleys likely prevented gene flow among populations, thus making them highly divergent even over short geographical distances.

In particular, the higher level of haplotype diversity observed in the central portion of the Maritime Alps (corresponding to the Argentera-Mercantour Group) suggests a long-time isolation of this area from the rest of the range. The inferred long isolation of these populations is also supported by their within-region genetic pattern. Besides being genetically most divergent, this clade exhibits a more structured distribution of haplotypes, compared to other clades. We could argue that the persistence in this refuge area was high during Pleistocene glacial and interglacial phases, as revealed by our Bayesian skyline plots. This scenario is congruent with the result of species distribution models, paralleling the biogeographic pattern of other species in this area, especially plants, during Pleistocene glaciations [18, 19, 21, 23, 24, 26].

Populations of *V. jugorum* inhabiting the northern part of the SW-Alps, where habitat suitability was strongly reduced during LGM, possibly persisted in spatially restricted refugial areas and unglaciated mountain peaks (‘nunataks’) in the interior of the Pleistocene ice shields. These scattered ice-free refugia, probably close to the sites of current occurrence, contributed to the long-term persistence of geographically isolated lineages, enabling the accumulation of among-populations genetic differentiation. The microclimatic heterogeneity of these refugia, in which topographic complexity may cause large temperature differences within short distances [19], might allow the populations to persist locally in regionally adverse conditions outside their LGM bioclimatic ranges. Such topographic complexity may decouple the local climatic conditions from those at the resolution used by species distribution model, resulting in potentially unpredictable bioclimatic suitability in these areas by the coarse-resolution of LGM projections.

Overall, it seems likely that the populations of *V. jugorum* survived in situ via short altitudinal shifts, migrating along the altitudinal gradient to follow their climatic optimum (‘*long-term stability hypothesis*’, sensu [16]), and remained isolated, causing the basal split observed in the phylogenetic tree. This pattern had previously been observed in some endemic plants of the SW-Alps [18, 19], but, to the best of our knowledge, it has rarely been observed in animals [75].

According to our phylogenetic reconstruction, the initial diversification of the lineages of *V. jugorum* traced back to the beginning of last Pleistocene glaciations (ca. 0.87 Mya), and continued over the entire glacial period. However, this prediction should be considered as a broad estimate, given the uncertainty associated. During this period, the extent of the ice shields would have caused an overall contraction of the wide ancestral distribution, prompting the species to find refuge in the southern, main refuge of Maritime Alps, at the periphery of the Pleistocene glaciers and in isolated refugia scattered across the species’ distribution. The following repeated cycles of glaciations likely have driven haplotypes divergence in refugial areas. In fact, the split between the Cottian and the Peripheral Group occurred ca. 0.62 Mya, while the divergence within the three main groups occurred principally during Riss and Würm glacial periods. However, the Mediterranean climatic mitigation effect, the high topographic heterogeneity and the relatively steep relief of this sector [22, 81], likely sheltered Maritime and Ligurian Alps from the impacts of the Pleistocene glaciers, minimizing populations and haplotypes extinction. Ancient divergence and local survival in microclimatic refugia are supported by the divergent and private haplotype (V9) found at Col de Vars (CV), in a region with a very low level of current habitat suitability in the northernmost portion of the range (see also in Mammola et al. [29]). In fact, this divergent haplotype could be the remnant of a former, isolated, wider population. This pattern has been previously suggested for some SW-alpine plants, in which the separation of the major clades occurred during the Early/Mid Pleistocene border, and the repeated glacial and interglacial periods drove the marked current intraspecific differentiation [18, 82].

Despite diversification by isolation seems to be the main pattern in affecting the genetic structure of *V. jugorum*, the genetic affinities between the population at Grande Sèolane and the remaining populations of the Peripheral Group, sharing one haplotype (V11), suggested that gene flow possibly occurred in the last 50,000 years. Tentatively, the establishment of corridors of suitable habitat at lower elevation between Provence and Maritime Alps could have promoted a recent colonization of the area of Grande Séolane by south-eastern lineages.

Future forecasts based on different emission scenarios showed significant shifts in the bioclimatic range, both towards higher latitudes and altitudes. This confirms our preliminary estimations [32], supporting the Endangered status for this species [33]. Because of its limited dispersal ability, *V. jugorum* may be unable to track the rapid shifts in its future bioclimatic niche, therefore remaining trapped within its current geographical range, which is expected to become largely unsuitable over the next few decades.

Our predicted changes in habitat suitability are expected to have a profound impact on the genetic diversity of the species. We predict that some of the southern and western peripheral portions of the range will remain isolated, due to a general loss of suitable areas in the Ligurian Alps, as well as to the appearance of isolated patches in the Provence Alps.

Such isolation processes may further deepen the divergence among populations. Moreover, depending on the strength of the climate change, it might also result in a substantial reduction of the current genetic diversity in the populations, which poses concerns to their long-term survival.

In particular, populations of the Ligurian Alps, of Serrière de la Lombarde in the Maritime Alps, and of Grande Séolane in the Provence Alps, are likely to go extinct according to the worst future climatic scenario. Local extinction would lead to the potential loss of haplotypes, resulting in loss of reservoirs of genetic variation and in genetic impoverishment of the species. Loss of genetic diversity is considered extremely detrimental from a conservation point of view, since low levels of diversity are generally correlated with reduced adaptive potential of the species [83]. We regard such peripheral populations as the most threatened by the ongoing climatic change.

Climate change could lead to an increase in suitable habitat for *V. jugorum* towards the NW-Alps. However, the pace at which suitable habitats are shifting, the low dispersal ability of the species (supported by the high population structuring and limited gene flow even between geographically close populations) as well as the fragmented distribution of screes, will likely hamper the possibility to reach newly available areas, suggesting that local extinction is more likely than migration.

## Conclusion

Combining species distribution modelling and mitochondrial phylogeography, our study explores the response of *Vesubia jugorum* to past, present and future climate changes. Our considerations have implications for conservation genetics, highlighting the role of the transboundary protected area of Parco Naturale Alpi Marittime and Parc National du Mercantour in promoting conservation efforts for this species. Indeed, the areas currently under protection facilitated the long-term survival of populations of *Vesubia jugorum* throughout past climatic oscillations, and they still support most of the current genetic variation. In this context, the preservation of genetic diversity in these areas is required for achieving a long-term conservation of the species.

### Supplementary Information

Additional File 1.

Additional File 2.

Additional File 3.

Additional File 4.

Additional File 5.

## Data Availability

All data generated or analysed during this study are included in this article and in its additional files. The ODMAP report of SDM parameters and assumptions for this study is available as Additional File 1. All the COI sequences have been deposited in GenBank NCBI as attached to the Additional File 2 (accession numbers from OR226670 to OR226717).
