# Supplementary material for: Combining distribution modelling and phylogeography to understand present, past and future of an endangered spider
Source: BMC Ecol Evol. 2024 Aug 5;24:106. doi: 10.1186/s12862-024-02295-2 (PMC11299272; doi:10.1186/s12862-024-02295-2)
Supplement: Supplementary file 3 — Additional File 3. [file 12862_2024_2295_MOESM3_ESM.docx]

**Table S3.** Analysis of Molecular Variance (AMOVA) grouping sequences by clades and sampling localities within clades.

| **Source of variation** | **Degrees of freedom** | **Sum of squares** | **Variance components** | **Fixation indices** | **Percentage of variation** |
| --- | --- | --- | --- | --- | --- |
| Among clades | 2 | 295.438 | 9.36008 | Fct = 0.76239  (p < 0.0001) | 76.24 |
| Among sampling localities within caldes | 10 | 96.375 | 2.56010 | Fsc = 0.87758  (p < 0.0001) | 20.85 |
| Within sampling localities | 25 | 12.500 | 0.35714 | Fst = 0.97091  (p < 0.0001) | 2.91 |
| Total | 47 | 404.312 | 12.27732 |  |  |
