# Supplementary material for: Combining distribution modelling and phylogeography to understand present, past and future of an endangered spider
Source: BMC Ecol Evol. 2024 Aug 5;24:106. doi: 10.1186/s12862-024-02295-2 (PMC11299272; doi:10.1186/s12862-024-02295-2)
Supplement: Supplementary file 4 — Additional File 4. [file 12862_2024_2295_MOESM4_ESM.docx]

**Table S4.** COI pairwise genetic distances shown as % uncorrected *p*-distance (± standard deviation) within and between sampling localities of *Vesubia jugorum*. Abbreviations of the sampling localities are explained in Fig. 2 and in Table 1.

|  | **CC** | **VDC** | **CDC** | **CM** | **CV** | **OS** | **BDA** | **GSL** | **MBG** | **PSA** | **SL** | **VAG** |
| --- | --- | --- | --- | --- | --- | --- | --- | --- | --- | --- | --- | --- |
| **CC** | 0.3 ± 0.1 |  |  |  |  |  |  |  |  |  |  |  |
| **VDC** | 1.1 ± 0.3 | 0.1 ± 0.1 |  |  |  |  |  |  |  |  |  |  |
| **CDC** | 2.4 ± 0.4 | 2.6 ± 0.4 | 0.1 ± 0.1 |  |  |  |  |  |  |  |  |  |
| **CM** | 2.1 ± 0.4 | 2.3 ± 0.4 | 0.6 ± 0.2 | 0.0 ± 0.0 |  |  |  |  |  |  |  |  |
| **CV** | 2.2 ± 0.4 | 2.4 ± 0.4 | 0.7 ± 0.2 | 0.6 ± 0.2 | 0.0 ± 0.0 |  |  |  |  |  |  |  |
| **OS** | 2.1 ± 0.4 | 2.3 ± 0.4 | 0.7 ± 0.2 | 0.0 ± 0.0 | 0.6 ± 0.2 | 0.1 ± 0.1 |  |  |  |  |  |  |
| **BDA** | 2.1 ± 0.4 | 2.5 ± 0.4 | 1.9 ± 0.4 | 1.8 ± 0.4 | 1.5 ± 0.3 | 1.8 ± 0.4 | 0.0 ± 0.0 |  |  |  |  |  |
| **GSL** | 2.5 ± 0.4 | 2.9 ± 0.5 | 2.2 ± 0.4 | 2.0 ± 0.4 | 1.9 ± 0.4 | 2.1 ± 0.4 | 0.6 ± 0.3 | 0.1 ± 0.1 |  |  |  |  |
| **MBG** | 2.3 ± 0.4 | 2.5 ± 0.4 | 1.9 ± 0.4 | 1.8 ± 0.4 | 1.6 ± 0.3 | 1.9 ± 0.4 | 0.3 ± 0.1 | 0.6 ± 0.2 | 0.0 ± 0.0 |  |  |  |
| **PSA** | 2.4 ± 0.4 | 2.8 ± 0.5 | 2.1 ± 0.4 | 2.0 ± 0.4 | 1.9 ± 0.4 | 2.0 ± 0.4 | 0.5 ± 0.2 | 0.1 ± 0.1 | 0.5 ± 0.2 | 0.0 ± 0.0 |  |  |
| **SL** | 2.4 ± 0.4 | 2.8 ± 0.5 | 2.1 ± 0.4 | 2.0 ± 0.4 | 1.9 ± 0.4 | 2.0 ± 0.4 | 0.5 ± 0.2 | 0.1 ± 0.1 | 0.5 ± 0.2 | 0.0 ± 0.0 | 0.0 ± 0.0 |  |
| **VAG** | 2.2 ± 0.4 | 2.5 ± 0.4 | 2.0 ± 0.4 | 1.9 ± 0.4 | 1.9 ± 0.4 | 1.9 ± 0.4 | 0.2 ± 0.1 | 0.5 ± 0.2 | 0.1 ± 0.1 | 0.4 ± 0.2 | 0.4 ± 0.2 | 0.0 ± 0.0 |

|  | **Argentera-Mercantour** | **Cottian** | **Peripheral** |
| --- | --- | --- | --- |
| **Argentera-Mercantour** | 0.7 ± 0.2 |  |  |
| **Cottian** | 2.3 ± 0.4 | 0.4 ± 0.1 |  |
| **Peripheral** | 2.5 ± 0.4 | 1.9 ± 0.3 | 0.3 ± 0.1 |
