# Supplementary material for: Combining distribution modelling and phylogeography to understand present, past and future of an endangered spider
Source: BMC Ecol Evol. 2024 Aug 5;24:106. doi: 10.1186/s12862-024-02295-2 (PMC11299272; doi:10.1186/s12862-024-02295-2)
Supplement: Supplementary file 5 — Additional File 5. [file 12862_2024_2295_MOESM5_ESM.docx]

**Table S5.** Eigenvalues and proportion of total variance explained by the first four axes, derived from the principal component analysis of climate data for our study area.

| **Axis** | **Eigenvalue** | **Percentage of variance** | **Cumulative percentage of variance** |
| --- | --- | --- | --- |
| PC1 | 12.48 | 62.39 | 62.39 |
| PC2 | 3.75 | 18.75 | 81.14 |
| PC3 | 1.47 | 7.34 | 88.48 |
| PC4 | 1.29 | 6.46 | 94.94 |
